# How to write a paper for a Journal

**DOI:** 10.4103/0019-5413.30526

**Published:** 2007

**Authors:** James Scott

**Affiliations:** Journal of Bone and Joint Surgery - British Volume, 22 Buckingham Street, London, WC2N 6ET UK

Before considering preparing a paper for presentation to the Journal prospective authors should read the Journal very carefully and regularly in order to become familiar with the contents, style and all aspects of the presentation of the material. Authors must read the Guide to Authors carefully and visit the website where some aspects of the presentation of, particularly clinical material, are highlighted.

Each element of the paper should be considered carefully and separately to be sure that it has a beginning and an ending and that each sentence follows logically. Thus the introduction initially simply requires a sentence indicating the background to the study with no more than three or four references. This should be followed by a sentence describing what investigation was undertaken, what question was asked or what hypothesis tested. The design of the study should subsequently be described in simple terms with an outline of the basic method.

The materials (or patients) and methods section requires a simple statement of how many patients made up the study group, why this number of patients was chosen and whether a power study was undertaken in order to indicate how many patients would be required to answer the question with statistical significance.

How were the patients chosen and what were the inclusion and exclusion criteria? If the study required randomization, how was this achieved? What tests were used? What outcome scores were chosen and why? Have these tests and scores been validated? If new tests or scores are being proposed have the appropriate validation studies with inter- and intra-observer errors been undertaken? It should be remembered that many outcome scores, such as the Mayo Elbow performance score, have not been validated but are in common usage. Care should be taken if scores have been modified with clear identification of how and by whom. Appropriate references for the tests and scores are required. What measurements were undertaken and are the units in which they are recorded appropriate? It is important to identify who undertook the measurements or tests or outcome scores and whether they were blinded. A case control study requires a careful description of how the controls were chosen, with an appropriate description of how they matched the study group.

The numbers of patients studied must be clearly separated from the number of operations, with indication of bilateral cases. Care must be taken when using percentages. Those patients who are lost to follow-up or have died must be identified with indication of why they could not be traced and why they left the study. Are the results relating to those lost to follow-up included in any of the data? Is this appropriate?

A clear description of the period of time during which the study was undertaken is required with an indication as to why this was chosen. The outcome after spinal decompressive surgery may be known in the immediate postoperative period but 30 years or more may be needed to assess the outcome after the treatment of congenital dislocation of the hip.

Life tables and survival analysis with Kaplan-Meyer tables and confident intervals are required for the presentation of long-term outcome.

Finally, at the end of this section a statement needs to be made with regard to ethical approval for the study and informed consent.

The statistics section needs simply to identify the statistical methods with *P*-values and confidence intervals and the relevant tests for each. It should be borne in mind that *P*-values indicate statistical significance and confidence intervals clinical significance. A simple identification of statistical equations which have been used and from whom statistical advice has been sought should be added.

The results section should be straightforward and clearly presented in a readily understandable fashion, with the appropriate use of tables and figures including suitable legends. Information in general should not be duplicated in the text and the tables. Particular care should be taken if illustrative radiographs are used to ensure the appropriate number and quality and if they clearly show what they are meant to show.

In this section the numbers of patients, tests, outcome scores, length of follow-up etc, should match those described in the materials and method section.

The discussion should only relate to the central question of the study and not stray into consideration of other related issues. The results should support the conclusion. It is important not to include significant bias into the interpretation of the results. A simple description of how they fit into the current state of knowledge is usually all that is required. Although “further research is required” should usually be avoided, if a specific particular further research question is raised by these results it should be identified.

The references should be appropriately set out and should be up-to-date and inclusive without bias. This is often not easy. Thus for instance the sentence “The Ilizarov technique can be used to treat ununited tibial fractures” needs only one or two appropriate references. It should be borne in mind that many reviewers will undertake a limited literature search.

The title of the paper is extremely important and may include a sub-title. It is clearly essential to ensure that the title adequately reflects the investigation. Care should be taken when using the word “prospective” as it is often inappropriately used to describe a retrospective study of prospectively gathered data.

Particular attention should be given to preparation of the abstract. In many ways this is the most important part of the paper as clearly with the increasing use of search engines it is the part of the paper which is the most read. An abstract should be a very carefully prepared concise piece of prose simply describing the purpose of the study and the methods used, with a sentence outlining the results and where this fits into our knowledge. For the Journal of Bone and Joint Surgery the abstract need not be structured however, the Indian Journal of Orthopaedics stresses on a structured abstract. Three or more key words and level of evidence may be added if specifically asked by particular journal. A guide to the level of evidence is usually available in the instructions to the authors.

When preparing material for presentation authors should consider how relevant and original their study is, whether aspects of it are controversial and if it confirms or disproves previously accepted findings. The limitations of the study should be outlined at the end of the discussion, with indications of the strengths and weaknesses of the investigation.

Ultimately the reviewers of the paper or the Editorial Board will have to consider whether the information within each element of the paper has been concisely and appropriately set out and whether this piece of work will advance knowledge or change practice.

